# Using Quality Improvement Methodology to Standardize Doppler Acquisition in a Pediatric Cardiology Echocardiography Laboratory

**DOI:** 10.1097/pq9.0000000000000380

**Published:** 2020-12-28

**Authors:** Eunice Hahn, Michael Taylor, Nikki Duncan, Angela Statile, James Brown, Garick Hill, Christopher Statile

**Affiliations:** From the * The Heart Institute, Cincinnati Children’s Hospital Medical Center; and; † Department of Pediatrics, Cincinnati Children’s Hospital Medical Center.

## Abstract

In echocardiography, the Doppler principle allows the measurement of tissue velocities. Using the Bernoulli equation, velocities are translated to estimated pressure differences, which dictate the timing of cardiac interventions. Our echocardiography laboratory demonstrated variability in the Doppler interrogation of heart valves, leading to difficulties in comparison over time and study accuracy. To align with previously published quality metrics in echocardiography, our laboratory used quality improvement methodology to achieve measurable improvement in Doppler acquisition.

**Methods:**

We developed a standardized protocol for Doppler acquisition and translated it to a 20-point scoring system. We established a baseline over 4 months via random assessment of 2 first-time, normal studies per day. Interventions included standardizing the process for acquisition, education, visual tracking, and individual feedback.

**Results:**

The percentage of studies with a score of 16 or higher preintervention was 17%. The median score was 13.4. In total, we analyzed 407 studies, 173 pre- and 234 postintervention. Over a 4-month intervention period, the median score improved to 18.1, with 85% of studies achieving a score of 16 or higher. Special cause variation occurred after protocol distribution, education, and feedback.

**Conclusions:**

Our initiative demonstrated significant improvement in the Doppler interrogation of cardiac structures using a measurable scoring system and a concrete goal of incorporating 20 areas of Doppler assessment in normal studies. Our next step is to spread this assessment to abnormal studies, thus developing consistency in evaluating all studies throughout the laboratory.

## INTRODUCTION:

The American Society of Echocardiography has promoted quality in echocardiographic assessment, with the publication of guidelines for echocardiography^1–5^ and recommendations for continuous quality improvement.^6,7^ Nationally, quality metrics exist for the echocardiographic assessment of congenital heart disease.^8^ However, despite these metrics, variability in approach to image acquisition has been reported nationally.^9^

The Doppler principle is based on the phenomenon whereby the frequencies of transmitted and observed waves change from a moving acoustic source relative to an observer.^10,11^ In echocardiography, this principle allows the measurement of blood and tissue velocities.^10,11^ This velocity can then be used in equations such as the simplified Bernoulli equation, which can estimate ventricular pressure or pressure differences across obstructed orifices.^10,11^ Criteria for intervention are based on these pressure differences.^12–14^ Doppler is a critical component of echocardiography, allowing for cardiac hemodynamic assessment without invasive testing.

A review of our echocardiography laboratory practices demonstrated a variable assessment of Doppler interrogation of the heart valves. This variability led to difficulty comparing valves over time and ambiguity about whether the maximum or appropriate Doppler assessment had been successfully achieved. Additionally, there was a patient-related event related to the inaccurate evaluation of aortic stenosis. These events, coupled with recent quality improvement initiatives by the American College of Cardiology (ACC), led to our initiative to improve Doppler assessment.^15,16^

Our initiative sought to implement a standardized process for Doppler acquisition in our laboratory.^5,14,17–19^ Though a prior echo protocol existed, it did not address specific guidelines for Doppler assessment. Our vision was that a protocol tested first on normal echocardiograms would ultimately facilitate a systematic assessment of all studies, allowing for comprehensive, comparable, and accurate assessment over time.

We developed a method to analyze our current baseline in Doppler assessment and then used quality improvement methodology to increase studies with adequate Doppler imaging as assessed by a scoring system. Specifically, we aimed to increase the percentage of studies with adequate Doppler assessment from a baseline of 17%–80% and increase the median score from 13 to 16 (out of 20) by June 30, 2018.

## METHODS

The echocardiography laboratory at Cincinnati Children’s Hospital Medical Center is part of the interdisciplinary Heart Institute and performs more than 13,000 studies each year. Thirteen attending physicians and 15 sonographers staff it. It provides acquisition and interpretation for echocardiograms performed at the primary base campus location, 6 affiliated campuses, 8 regional clinics, and 4 external nurseries. Images are entered and accessed via the Syngo Dynamics reporting system (Siemens Healthineers, Erlangen, Germany).

**Fig. 1.**
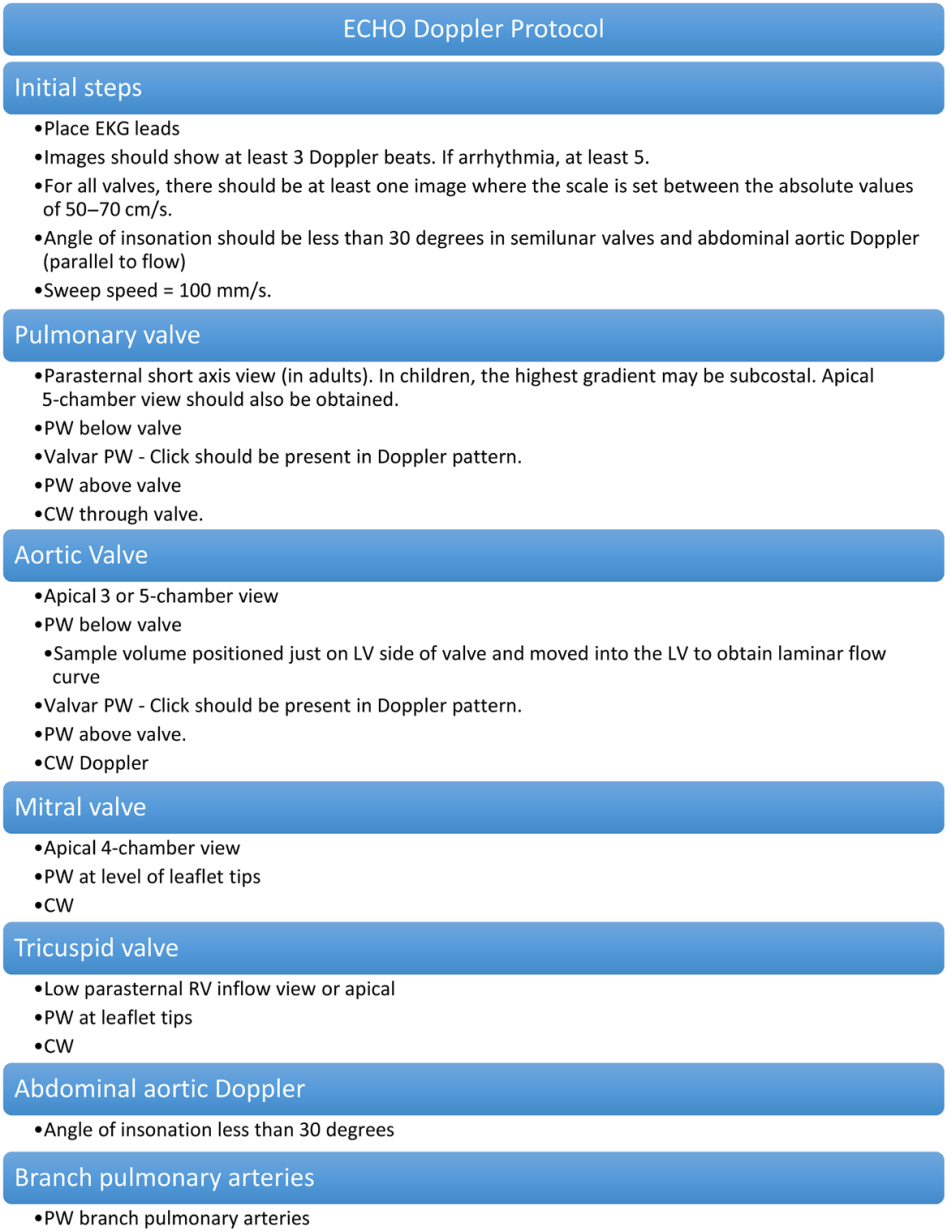
Standardized Doppler protocol. CW, continuous-wave Doppler; PW, pulsed-wave Doppler.

**Fig. 2.**
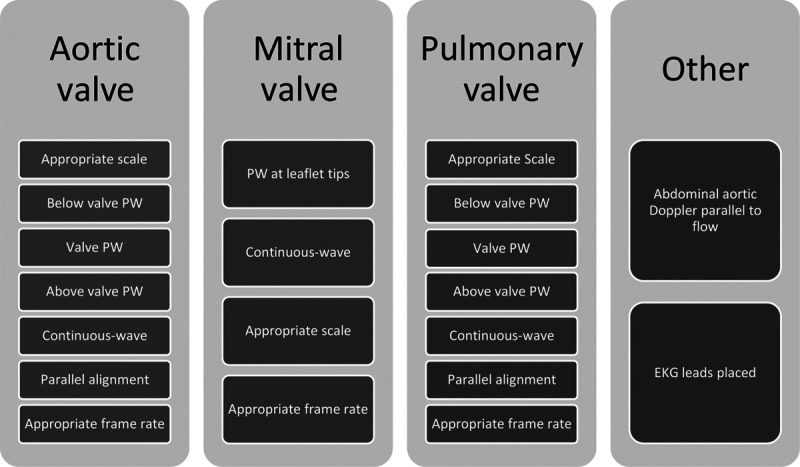
The Doppler scoring system. Each component received one point for a total of 20 points. PW, pulsed-wave Doppler.

To support the comprehensive Doppler interrogation of heart valves, a group of key stakeholders, including Cincinnati Children’s Hospital Medical Center physicians, sonographers in echocardiography, and quality improvement experts, developed a standardized protocol for Doppler acquisition based on literature review^5,14,17–19^ and a consensus of standard practice (Fig. 1). We focused on normal studies as the first step in our initiative, with the philosophy that repetition of a normalized process over time would be necessary before spreading our effort to abnormal studies. A short and a long protocol were available for ease of use with comparable content. These protocols were then translated into a 20-point scoring system to allow for the initiative’s concrete aim and to track the initiative over time (Fig. 2). Each component of the protocol counted as one point upon completion, with a maximum possible score of 20 points. We suggested optimal views to assess each valve. We considered the angle of insonation adequate if it was less than 30 degrees. There had to be at least one image of valves with a scale between 50 and 70 cm/sec, to allow for assessment of valve regurgitation per American Society of Echocardiography standards and comparisons of regurgitation at a similar Nyquist over time.^17–19^

This project fell within the Institutional Review Board’s guidance for quality improvement projects, and thus informed consent was waived.

### Improvement Implementation

**Fig. 3.**
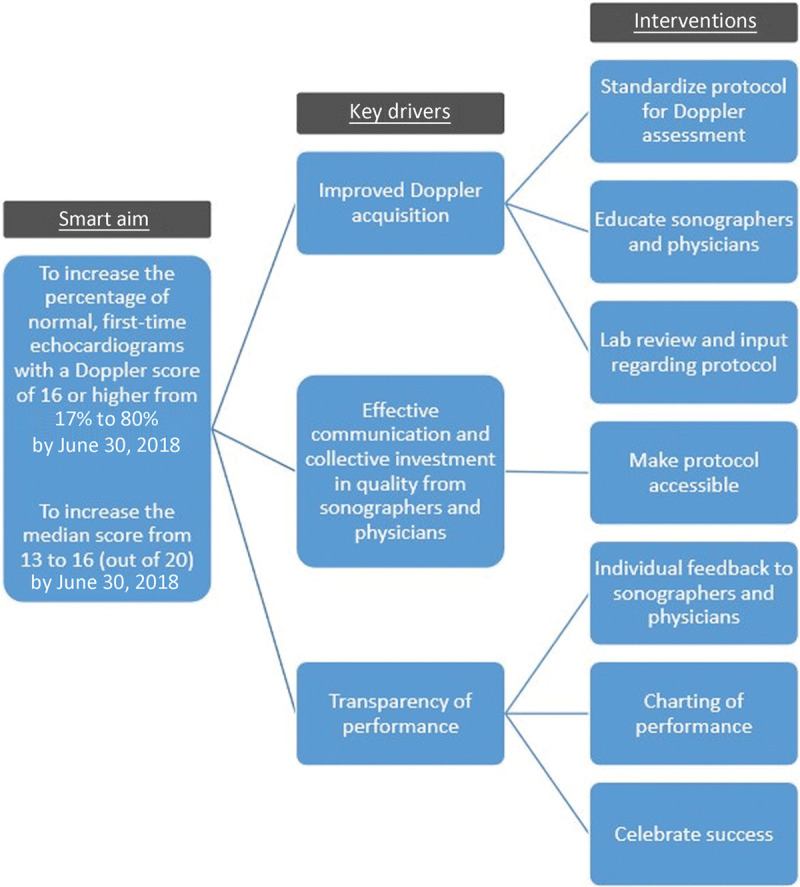
Key driver diagram showing our smart aim, key drivers, and interventions designed around the key drivers.

Our key drivers to achieve our aim included (1) improved Doppler acquisition through standardization, (2) effective communication, and (3) transparency of performance to guide continual improvement (Fig. 3). We tested and implemented changes using plan-do-study-act cycles.

### Improved Doppler Acquisition

Before the intervention, there was variability in the scale used to assess valves and the level at which valves were interrogated. The color scale was adjusted to 50–70 cm/sec to allow for uniformity in valve regurgitation assessment. We set the machine default to this level. We provided labeled diagrams to illustrate the appropriate levels to assess a valve in the subvalvular, valvar, and supravalvar region.

### Effective Communication

The protocol was initially tested and modified with 2 sonographers. At meetings, we educated sonographers and physicians regarding the protocol and distributed it. We obtained collective feedback regarding the protocol and made clarifications accordingly. The protocol was also laminated and placed on the echocardiography machines. In the first 3 days of protocol implementation, the sonographers filled out a survey assessing whether the protocol could be completed and, if not, the reasons why.

### Transparency of Performance

We tracked improvement by control charts, communicated the progress via email, and posted a chart in the echocardiography laboratory. Once the protocol was spread widely, we provided education and feedback regarding the most frequently missed areas by visual aids, including still images of the appropriate assessment. We analyzed each sonographer’s performance and gave individual feedback regarding the areas that required improvement. We celebrated when we achieved our goal.

### Development of Aim and Outcome Measures

Randomly, we conducted an assessment of the 2 first-time, normal studies per day, if available. To establish a baseline, we evaluated 173 studies over 4 months. The primary outcome measures were an adequate median score, defined as 16, and the percentage of studies that achieved this score. We chose this score (equivalent to 80%) to allow for uncontrolled patient factors. During the baseline period, the percentage of studies that achieved a 16 or higher score was 17%, and the median score was 13.4. Our SMART aim was to increase the percentage of studies with a Doppler score of 16 or higher from 17% to 80% and increase the median score from 13 to 16 (out of 20) by June 30, 2018.

### Analysis

We tracked the outcome measures over time using statistical process control charts.^20^ A P chart is a control chart that plots attribute data over time. Upper and lower control limits represent the natural variation in the system. Common cause variation (variation inherent to the system) occurs randomly within the control limits. Special cause variation, or a change that occurs external to how a system operates typically, can occur because of unplanned external influencers or planned interventions. The statistical theory defines rules for special cause. We relied on three rules to detect special cause variation:

1. A single point outside the control limits (*P* = 0.0015)
2. Six consecutive points trending up or trending down (*P* = 0.00278), or
3. Eight or more consecutive points above or below the centerline (*P* = 0.0078).

## RESULTS

This initiative incorporated 2 randomly selected, first-time, normal echocardiograms per day performed between October 1, 2017, and June 30, 2018. The study included a 4-month preintervention period and a 4-month intervention period. In total, we analyzed 407 studies and excluded no studies.

**Fig. 4.**
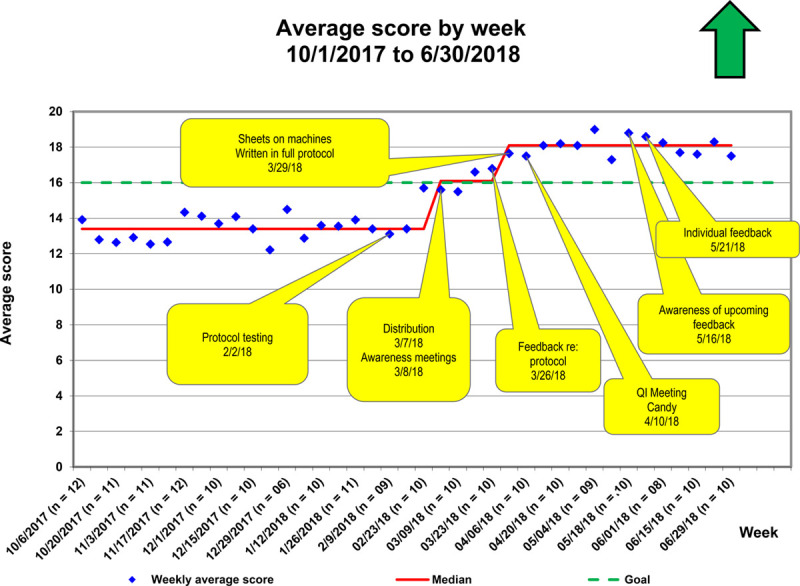
P chart showing average score by week. Interventions are labeled in yellow. The red line denotes the running median value. The goal is shown with a green dotted line. The figure shows that over time, with the implementation of interventions, there was an improvement of the score until the goal was met.

**Fig. 5.**
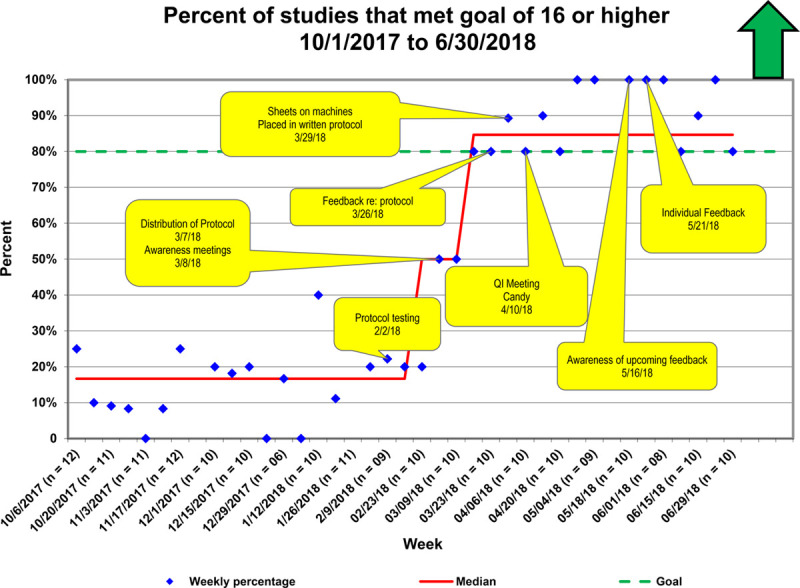
P chart showing the percentage of studies that met a goal of 16 or higher. Interventions are labeled in yellow. The red line denotes the running median score. The goal of 80% studies meeting goal is denoted with a green dotted line. The figure shows that over time, with the implementation of interventions, there was an improvement in the percentage of studies that met goal.

**Fig. 6.**
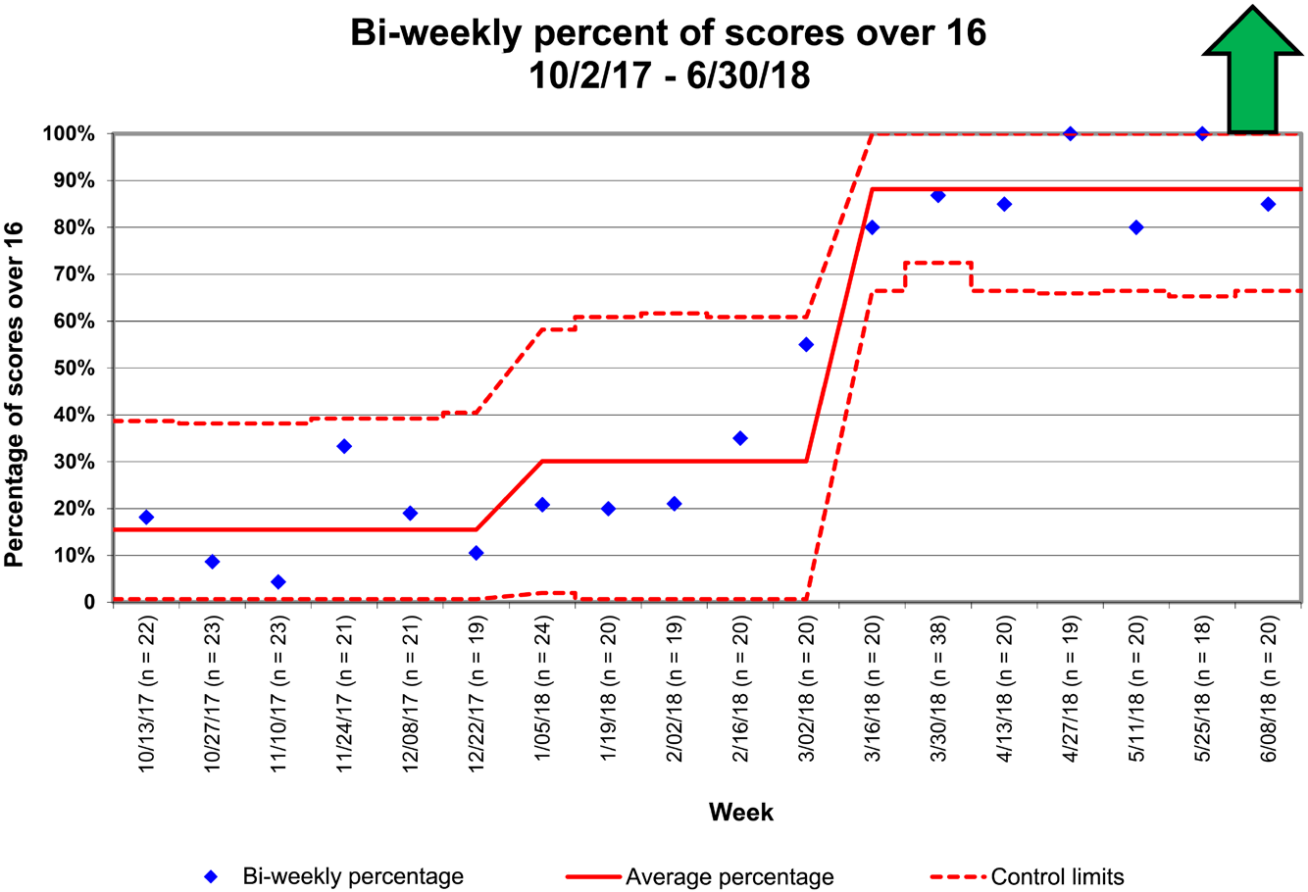
Control chart of the percentage of studies that met a goal of 16 or higher demonstrated bi-weekly. The solid red line denotes the running mean value. The dotted red lines represent control limits. The figure shows that over time, with the implementation of interventions, there was an improvement in the running mean.

The first special cause variation occurred after distributing the standardized protocol via awareness meetings (Figs. 4–6). The second special cause variation occurred after collective feedback regarding the protocol, with the incorporation of the Doppler protocol into the standard echocardiographic assessment protocol for the laboratory. Before the intervention, the top 4 items not performed in the protocol were a supravalvar aortic pulsed-wave Doppler (84% omitted), mitral valve continuous-wave Doppler (83% omitted), supravalvar pulmonic pulsed-wave Doppler (50% omitted), and appropriate abdominal aortic Doppler (47% inadequate). Following the intervention, these areas showed improvement: aortic pulsed-wave Doppler (36% omitted), mitral valve continuous-wave Doppler (13% omitted), supravalvar pulmonic pulsed-wave Doppler (13% omitted), and appropriate abdominal aortic Doppler (24% inadequate).

Over the 4-month intervention period, the median score improved from 13.4 to 18.1, with 85% of studies achieving a 16 or higher score. We sustained achievement above goal for 3 months. The control chart demonstrated that over time, there was an improvement in achieving adequate Doppler assessment, with more stability in this process (Fig. 6).

## DISCUSSION

Doppler’s use in echocardiography has been heralded as a “noninvasive hemodynamic ultrasonography revolution” that occurred in the late 1970s and throughout the 1980s.^21,22^ The incorporation of Doppler into 2-dimensional echocardiography allowed surgical decisions to be based on noninvasive hemodynamic measurements, which benefited patients and granted interventionalists the time to move forward with transcatheter innovation.^21^ With the rapid advances in echocardiography, there has been concern regarding the variable acquisition processes in laboratories, making uniform and accurate interpretation difficult.^9,23^ Thus, there has also been a national drive to improve echocardiography laboratories’ quality, of which standardized study acquisition is a crucial component.^4–7,9,15,23^ We present a quality initiative in our laboratory that improved the consistency of Doppler acquisition, which translated into an improvement in Doppler assessment completeness from 17% to 85%.

We designed our QI intervention with consideration of patient, provider, and system-level factors. Our echocardiography laboratory faces multiple challenges daily, which are not unique to our institution. Multiple systemic factors can contribute to variability in practice, including lack of patient cooperation, available imaging windows, optimal study environment, emergent situations, and time. There are varying schedules for each of the sonographers in our laboratory, who may travel to 4 different hospitals and 15 outpatient locations in 3 states. Furthermore, our sonographers vary in experience and educational background and, thus, are accustomed to a variety of valvar assessment methods.

Despite these challenges, we used QI methodology to decrease the variability of our practice.^24^ We developed a new protocol that was standardized, efficient, and applicable to variable situations. The entire echocardiography laboratory provided input to achieve buy-in, and we worked together toward a common goal. We avoided a manager versus the managed-mentality of an overworked, often underappreciated group of sonographers by working together. Effective communication and education were performed through multiple venues (lectures, huddle sessions, email, and pamphlets) to accommodate variable schedules. Individualized sessions focusing on components missed by each sonographer were crucial for promoting aggregate improvement. Performance tracking was readily visible with control charts, and we celebrated the achievement of a collective goal.

There are several potential reasons that we were able to improve our Doppler acquisition. Standardization of Doppler acquisition with a quantifiable and visible assessment of adherence gave clear goals to an already skilled group. This standardization is consistent with recommendations from the ACC in achieving quality in cardiovascular imaging.^15,16^ Namely, this consensus statement states that “high-quality image acquisition depends on modality-specific processes, including specific protocols and sequences that optimize the likelihood that images are of sufficient diagnostic quality.”^15^ A crucial element to our success was to have the buy-in of our staff, with constant communication. This factor also aligns with ACC recommendations, highlighting that “achieving quality in cardiovascular imaging requires the sustained, coordinated efforts of many stakeholders.”^15^ Other centers can apply the keys to our success: (1) standardization of a process, (2) concrete goals, and (3) collective buy-in with coordination toward a common goal.

We are using lessons from this quality improvement initiative to sustain and improve compliance with the Doppler protocol. We were successful in improving some critical systematic challenges. For example, in any echo laboratory, there are time constraints for image acquisition. Each study is scheduled for completion in 60 minutes, including the collection of patient height and weight, scanning, measuring, and preliminary reporting. We now have separate personnel to collect patient height and weight, thus optimizing the image acquisition and reporting time. We have also configured our reporting system so that the tools to make first-time measurements are all in one place instead of clicking between screens. For efficiency, in certain situations, a sonographer may choose to skip multiple Doppler evaluations of a normal outflow tract to focus on acquiring other information, such as the anatomic images needed to complete a study. To mitigate possibly unmodifiable patient-specific factors (eg, uncooperativeness, skin conditions, limited echocardiography windows, type of heart disease, or the need for a limited study for a specific purpose), we are considering targeted protocols for specific age groups, uncooperative patients, or those with limited echocardiographic windows. To improve sustainability in the face of challenges with sonographer turnover and shortage, we implemented early education of the new protocol to any new hires and incorporated protocol adherence into the quality assessment. Our next step will be to spread this protocol to assess congenital heart disease, thus enabling consistent, comparable assessment over time and accuracy in identifying levels of obstruction.

Our initiative should be interpreted in the context of its limitations. Though most measures were objective in the score assessment, the adequacy of an angle of insonation or parallel Doppler is often based on reader judgment. We included this measure due to its importance in the Doppler principle, where Doppler interrogation parallel to flow achieves the most accurate assessment.^10^ This initiative was performed at a single center, though we are optimistic that other centers can apply this methodology. The score has not been validated at multiple institutions. Finally, we have limited data postintervention and will need to reassess the initiative’s sustainability.

## CONCLUDING SUMMARY

This QI initiative used quality improvement principles to design and implement a standardized protocol in the valvar assessment of patients using Doppler. We demonstrated significant improvements based on a measurable scoring system with a concrete goal incorporating 20 areas of Doppler assessment. Our next step is to spread this assessment to all abnormal studies. We hope that this process will improve accurate Doppler assessment for our patients, and others nationally.

## DISCLOSURE

The authors have no financial interest to declare in relation to the content of this article.
